# Identification of an immunodominant IgE epitope of Der f 40, a novel allergen of *Dermatophagoides farinae*

**DOI:** 10.1016/j.waojou.2023.100804

**Published:** 2023-08-01

**Authors:** Ze-Lang Cai, Shan Liu, Wei-Yong Li, Zi-Wen Zhou, Wan-Zhen Hu, Jia-Jie Chen, Kunmei Ji

**Affiliations:** a Department of Biochemistry and Molecular Biology, School of Basic Medical Sciences, Shenzhen University Medical School, Shenzhen 518060, China; b Shenzhen University General Hospital, Shenzhen 518060, China

**Keywords:** Dust mite allergy, Allergens, Immunodominant epitopes, Immunoglobulin E

## Abstract

**Background:**

House dust mites (HDMs), including *Dermatophagoides pteronyssinus* (Der p) and *Dermatophagoides farinae* (Der f) species, represent a major source of inhalant allergens that induce IgE-mediated anaphylactic reactions. HDM allergen identification is important to the diagnosis and treatment of allergic diseases. Here, we report the identification of a novel HDM allergen, which we suggest naming Der f 40, and its immunodominant IgE epitopes.

**Methods:**

The recombinant protein Der f 40 was expressed using a pET prokaryotic expression system and purified with Ni-NTA resins. IgE binding activity was evaluated by IgE-western blot, dot-blot, and ELISA. Mast cell activation testing was performed to assess the cellular effects of IgE binding in mouse bone marrow derived mast cells (BMMCs) expressing human FcεRI. IgE binding assays were performed with truncated and hybrid Der f 40 protein molecules to find immunodominant IgE epitopes.

**Results:**

A 106-amino acid (aa) recombinant Der f Group 40 protein (rDer f 40) was obtained (GenBank accession No. XP_046915420.1) as thiredoxin-like protein. Der f 40 was shown to bind IgE from HDM allergic serum *in vitro* (9.68%; 12/124 in IgE-ELISA), and shown to promote the release of β-hexosaminidase from BMMCs dose-dependently when administered with HDM allergic sera. The Der f Group 40 protein was named Der f 40 and listed in the World Health Organization and International Union of Immunological Societies (WHO/IUIS) Allergen Nomenclature Sub-committee. IgE binding assays with Der f 40-based truncated and hybrid proteins indicated that IgE binding epitopes are likely located in the C-terminal region and dependent on conformational structure. The 76–106-aa region of C-terminus was identified as an immunodominant IgE epitope of Der f 40.

**Conclusion:**

A novel HDM allergen with robust IgE binding activity was identified and named Der f 40. An immunodominant IgE epitope of Der f 40 with conformational dependency was identified in the C-terminus (aa 76–106). These findings provide new information that may be useful in the development of diagnostic and therapeutic agents for HDM allergy.

## Introduction

House dust mites (HDMs), especially *Dermatophagoides pteronyssinus* (Der p) and *Dermatophagoides farinae* (Der f) species, are major sources of inhalant allergens and are allergenic to a majority of patients with allergic diseases.^1^^,^^2^ In-depth analyses of the full spectrum of HDM allergenic components are needed to further elucidate the mechanism of HDM allergy and to guide the development of diagnostics and immunotherapeutic vaccines.^3^

The quantity of HDM allergens that exist remains a key question in allergy research.^4^ HDM allergen nomenclature continues to be updated as new allergens are found. Thus far, 39 HDM allergen groups have been reported, but not all of the groups have been identified yet in Der p and Der f. The WHO/IUIS (World Health Organization and International Union of Immunological Societies) allergen database identifies only 31 Der p allergen groups and 36 Der f allergen groups (www.allergen.org). Component-resolved analysis studies have revealed that HDM-allergic patients exhibit distinct individual sensitization profiles with respect HDM allergen reactivity,^5^ and known HDM allergens are insufficient to clearly explain the observed profiles. Thus, further work remains to determine the sequences, biochemical characteristics, and structures of novel HDM allergens.

Advances in potential allergen discovery and identification methods have accelerated the identification and naming of HDM allergens, such as IgE-binding screening of potential allergens (natural or recombinant from a cDNA library), two dimensional (2D) proteomics, or high-precision genomics-based homologs.^6^ Early studies honed in on major HDM allergens, which came to constitute Group 1 and Group 2.^7^ In a study of 200 Chinese patients with Der p IgE reactivity, 89% had IgE reactivity against Der p 1 and 84% had IgE-reactivity against Der p 2.^8^ Using 2D SDS-PAGE (sodium dodecyl-sulfate polyacrylamide gel electrophoresis) and IgE western blotting, 4 novel HDM allergens (Der f 25, Der f 28, Der f 29 and Der f 30) from 17 IgE-reactive protein spots in 2D-separated proteins were identified.^9^ Additionally, in our previous studies, we showed that group 24 HDM allergens from Der f and Der p were ubiquinol cytochrome C reductase binding (UQCRB) protein homologs based on combined transcriptome and proteome analysis of the HDM allergome.^10^ Moreover, allergen homolog analysis of HDM genomes on a chromosome level showed that Der f 39 and Der p 39 are troponin C homologs; these allergens were recognized by IgEs from 7/76 and 5/87 subjects tested, respectively.^11^^,^^12^

Even with the methodological advances that have been attained, not all HDM allergens can be found clearly at one time or in high-throughput applications. HDM allergen screening is limited by the accuracy of HDM genome data, the integrity of HDM extraction, and the specificity of HDM-allergic sera. Allergen identification requires enough natural or recombinant protein candidates to perform IgE-binding activity assays.^13^ Identification of HDM allergens en masse has been limited by the large number of HDM allergen homologs, some being derived from natural extract and others being recombinant proteins. In a previous study, we showed that 37 candidate homologs could be filtered out using homology analysis, and then cloned 8 remaining candidate homologs of interest and expressed them for identification of allergenicity testing. Two of those remaining candidates, now known as Der f 37 and Der f 39, were confirmed to be HDM allergens. The previous study pointed out 21 potential allergen homologs to be identified in Der f.^11^

The aim of this study was to identify whether Der f 40 is a novel HDM allergen. We cloned, expressed, and purified Der f 40 recombinant protein. We subjected the resultant recombinant Der f 40 (rDer f 40) to serum IgE binding assays and performed IgE binding experiments, including western blots, dot blots, and ELISAs, to identify the immunodominant IgE epitope of Der f 40. Hybrid proteins were produced and analyzed because the hybrid protein method has been shown to be helpful for screening and identifying conformational epitopes of allergens.^12^^,^^14^ The thus characterized Der f 40 allergen was submitted to the WHO/IUIS database. The identification of a novel Der f 40 allergen would be clinically useful for the diagnosis and treatment of HDM induced allergic diseases.

## Materials and methods

### Human serum samples

Serum samples were collected from 124 HDM allergic patients (57 males and 67 females; age range, 18–58 years) and 50 non-allergic individuals (23 males and 27 females; age range, 18–72 years) at Peking Union Medical College Hospital (Beijing, China). IgE binding was quantitated with an ImmunoCAP allergen detection system (Pharmacia Diagnostics, Uppsala, Sweden). A binding level ≥3 was considered to be indicative of IgE specificity for an HDM allergen group. All procedures involving human participants were conducted according to the ethical standards of the committee.

### Animals

The humanized-IgE/FcεR1 model mice were purchased from Cyagen (Santa Clara, CA). All animal care and experimental procedures were carried out according to protocols approved by the Animal Care and Use Committee of Shenzhen University Medical School.

### Cloning and expression of Der f 40

Der f total RNA was extracted with a Minibest Universal RNA Extraction Kit (TaKaRa, Tokyo, Japan) and reverse transcribed rapidly into cDNAs with an Evo M-MLV RT Mix Kit (Accurate Biology, Changsha, China). Open reading frame (ORF) DNA was amplified by reverse transcriptase (RT)-polymerase chain reaction (PCR) with Der f 40-specific primers designed based on the latest Der f genome data (NCBI Accession no. PRJNA811591; ID 811591). The forward primer was 5′-ATGGTTCACAAAGTTACTGA-3′ and the reverse primer was 5′-TTAACTGTATTGTTGAATGG-3’. After confirmation by DNA sequencing, the Der f 40 ORF DNA was cloned into a pET-His vector to express the recombinant protein in *Escherichia coli* BL21(DE3)lysS. The system was induced with 1 mM IPTG for 4 h at 25 °C and rDer f 40 was expressed in the soluble fraction. The rDer f 40 protein was purified by Ni-NTA chelating, anion-exchange, and gel filtration chromatography.

### Sequence alignment and protein structure modeling

The amino acid (aa) sequence of Der f 40 and homologous thioredoxin (TRX) proteins from other species were aligned in Cluster Omega software.^15^ A homology model of the structure of Der f 40 was constructed according to the analysis of the Swiss Model Repository^16^ using the Mala s 13 crystal structure, based on a *Malassezia sympodialis* template (PDB 2J23), and presented in Pymol software (version 2.3.0).^17^

### TRX activity measurements

Enzymatic activity of rDer f 40 was evaluated by measuring catalytic reduction of insulin. In 3-ml reaction mixtures containing 50 mM Tris HCl (pH 7.5), 1 mmol EDTA, 2 mM DTT, 0.17 mM bovine insulin, and 5 μM rDer f 40, absorbance at a wavelength of 690 nm was monitored by spectrometry. Reaction mixtures without recombinant protein were used as negative control samples.

### IgE ELISA

Microtiter plate wells were coated with 1 μg of protein overnight at 4 °C. The next day, 5% (w/v) bovine serum albumin (BSA) in phosphate buffered saline (PBS) containing 0.05% Tween 20 was applied for 2 h at 37 °C to block. Then, the plates were incubated with serum from HDM allergic patients (1:5 dilution) for 2.5 h at 37 °C. After washing, mouse anti-human IgE (Fc)-horse radish peroxidase (HRP) (Southernbiotech, Cat. 9160-05, 1:2000) was added for 1 h at 37 °C. Absorbance at 450 nm was measured by a microplate reader (Bio-Rad, USA) after adding the HRP substrate 3,3′,5,5′-tetramethylbenzidine and stopping the reaction with H_2_SO_4_. IgE-ELISA results with a positive/negative value (P/N) > 2.1 were considered positive, where P/N is the ratio of optical density values of positive result samples and negative result samples. All tests were performed in triplicate.

### IgE western blot

Protein samples were separated by 15% SDS-PAGE and electrophoretically transferred to polyvinylidene fluoride membranes (Millipore, USA), which were blocked with 5% BSA (diluted in Tris buffered saline with 0.1% Tween20 buffer) overnight at 4 °C. HDM allergic serum and healthy serum samples (1:5 dilutions) were incubated with the membranes for 2.5 h at 37 °C, separately. Mouse anti-human IgE (Fc)-HRP (Southernbiotech, Cat. 9160-05, 1:1000) was added for 1 h at 37 °C after washing. The bands were visualized with a Pierce™ 3,3′ diaminobenzidine (DAB) substrate Kit (Thermo Scientific, Cat. 34002).

### IgE dot blot

Nitrocellulose membranes (Millipore, USA) were cut into 0.5 × 0.5-cm squares and loaded with protein samples, air-dried, blocked with 5% BSA for 2 h at 37 °C, and then incubated with the HDM allergic serum and healthy control serum samples (1:5 dilutions) for 2.5 h at 37 °C, separately. Mouse anti-human IgE (Fc)-HRP (Southernbiotech, Cat. 9160-05, and 1:1000 dilution) was added for 1 h at 37 °C after washing. Binding was visualized as brown precipitates on membranes by applying DAB substrate Kit (Thermo Scientific, Cat. 34002).

### Mast cell degranulation assay

Bone marrow derived mast cells (BMMCs) were isolated from the femurs of a humanized-IgE/FcεR1 BALB/c mice then cultured in RPMI-1640 with penicillin, streptomycin, fetal bovine serum, IL-3, and stem cell factor according to the previously described protocol.^18^ Flow cytometry detection of CD117 and FcεRI expression on cell surfaces after 4–6 weeks in culture confirmed ≥95% mast cell purity.

The BMMCs were subjected to a β-hexosaminidase release assay. Four individual Der f 40-sensitized HDM allergic patients' sera (in 1:8 dilutions) were incubated overnight; sera from healthy individuals served as negative controls. The next day, the cells were washed with Tyrode's buffer 3 times. The cells were incubated with different concentrations of rDer f 40 for 1 h at 37 °C. Supernatant and lysate samples were reacted with 4-nitrophenyl-N-acetyl-β-d-glucosaminide for 1 h at 37 °C and the reaction were stopped with carbonate buffer. Absorbance at a wavelength of 405 nm was determined using a multi-well plate reader.

### Expression and purification of rDer f 40-related proteins

Six Der f 40-derived overlapping epitope sequences were designed and subcloned into pET-DsbA prokaryotic expression vectors (miaolingbio, Wuhan, China) for recombinant protein expression in *E. coli* BL21(DE3) plysS. The following truncated forms of Der f 40 were employed: Der f 40-E1(aa 1–30), Der f 40-E2(aa 16–45), Der f 40-E3 (aa 31–60), Der f 40-E4 (aa 46–75), Der f 40-E5 (aa 61–90), Der f 40-E6 (aa 76–106). In addition, hybrid proteins that integrated human TRX protein sequences into Der f 40 were designed; the truncated and hybrid Der f 40 cDNA sequences were synthesized into proteins by Genescript Corporation (Nanjing, China). The expression and purification of the recombinant proteins were performed as described previously.^12^ The recombinant fusion proteins were in the soluble fraction of the cell lysate and purified by nickel affinity purification. The purified recombinant proteins’ concentrations were measured with Bradford assays (Sangon Biotech Co., Ltd). These recombinant Der f 40-related proteins were used to screen for IgE epitopes.

### Statistical analysis

Data are presented as means with standard deviations (SDs). Statistical analyses were conducted in Prism7 (GraphPad Software, La Jolla, CA). Differences between groups were determined by one-way analyses of variance (ANOVAs) followed by Dunnett's t tests for multiple comparisons. P values < 0.05 were considered significant.

## Results

### Identification of an allergenic TRX homolog in Der f

**Fig. 1:**
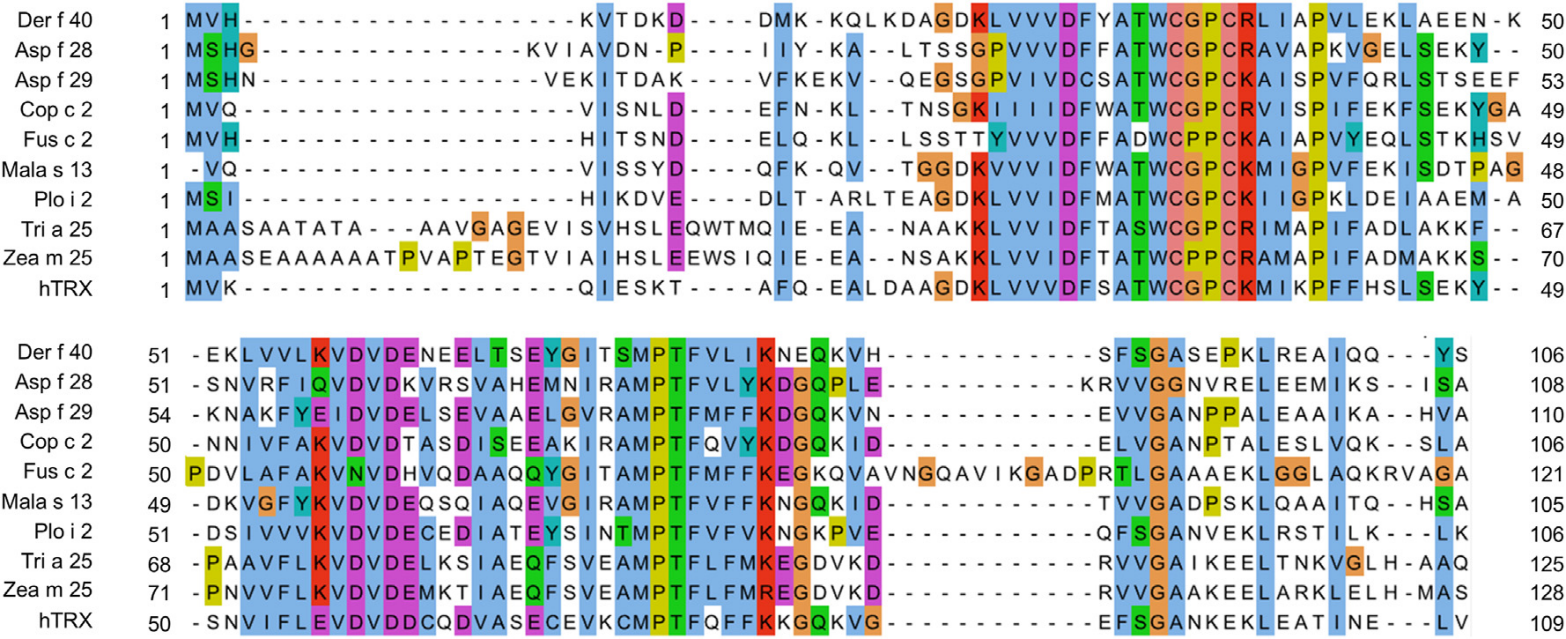
Multiple sequence alignment of aa sequences of Der f 40 and thioredoxin from other species generated in Clustal Omega software. Der f: *D. farinae*; Asp f: *Aspergillus flavus*; Cop c: *Coprinus comatus*; Fus c: *Fusarium culmorum*; Mala s: *Malassezia sympodialis*; Plo i: *Plodia interpunctella*; Tri a: *Triticum aestivum*; Zea m: *Zea mays*; hTRX: Human thioredoxin

**Fig. 2:**
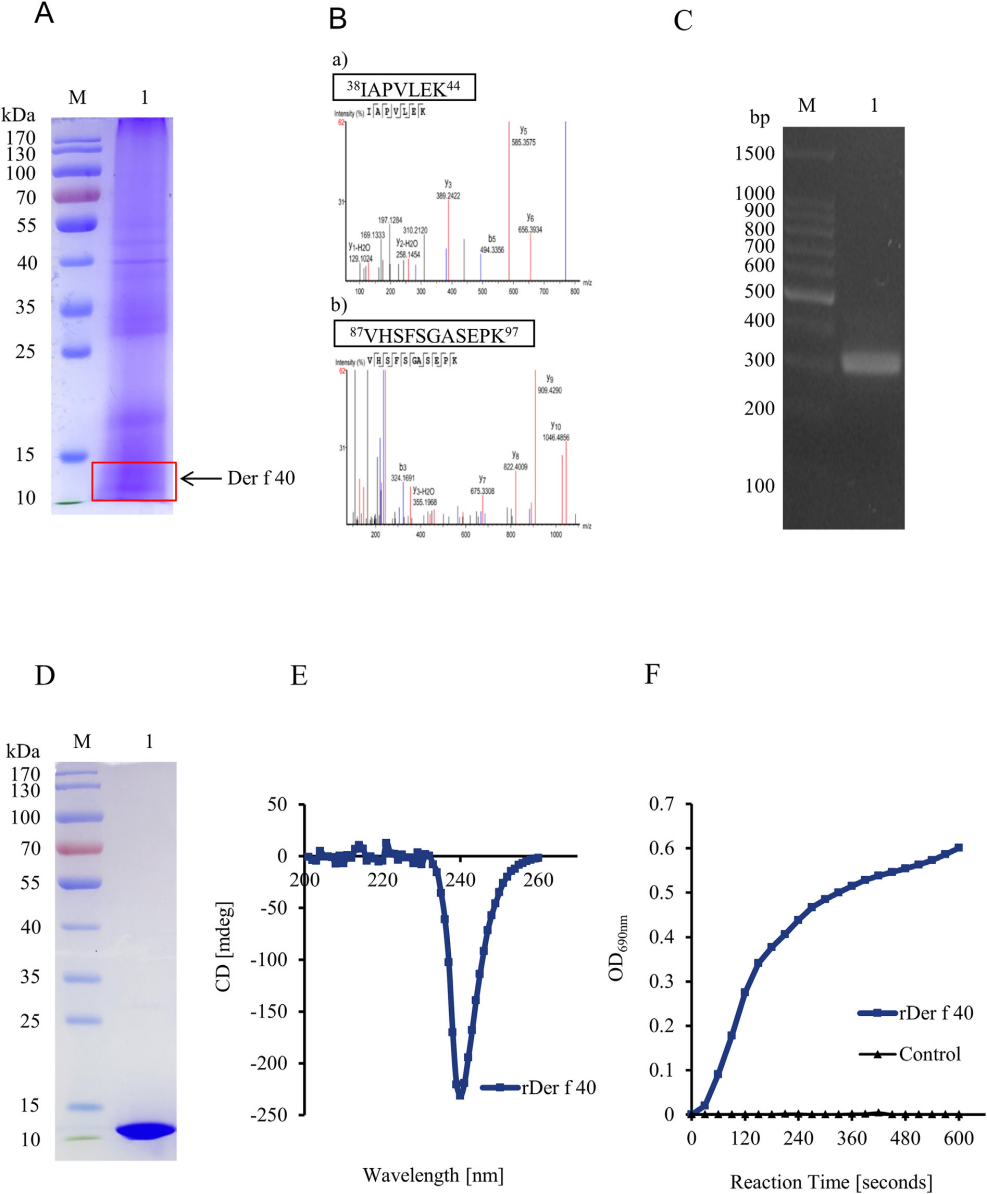
**Expression, purification, and enzymatic characterization of rDer f 40.** A. SDS-PAGE of Der f crude extract. The natural thioredoxin protein may be present at ∼12 kDa band (red box in Lane 1). B. LC/MS analysis of the ∼12 kDa band sample obtained from crude Der f extract. Two representative coverage peptides with the aa sequences ^38^IAPVLEK^44^ and ^87^VHSFSGASEPK^97^. C. Der f 40 ORF cDNA was amplified by RT-PCR using specific primers. M: DNA Marker, Lane 1: Der f 40 PCR results. D. SDS-PAGE of the purified rDer f 40 protein expressed in *E. coli*. M: Prestained protein marker, Lane 1: purified rDer f 40 protein. E. Far-ultraviolet (wavelength, 200–260) circular dichroism analysis of purified rDer f 40 at 25 °C. The CD spectrum CD spectrum shows that it is consistent with the secondary structure of Der f 40 protein predicted, mainly composed of α-helix. F. Insulin reduction assay of thioredoxin activity using rDer f 40

According to the previous work examining the remaining 21 allergen homologs in Der f, Der f TRX may be a candidate novel HDM allergen Der f 40. Analysis of homologous alignment of aa sequences from our Der f transcriptome (BioProject ID PRJNA512594; accession no. SDOV00000000) indicated that Der f TRX(GenbankNo. XP_046915420.1) shows 52% homology with Plo i 2 of *Plodia interpunctella* (GenbankNo. FR681573.1) (Fig. 1). A 12-kDa protein band from an SDS-PAGE gel of Der f crude extract was subjected to LC/MS analysis to identify the putative endogenous Der f 40 protein (Fig. 2A). A 22.64% coverage rate was achieved with representative results from 2 coverage peptides (aa 38–44 and aa 87–97) (Fig. 2B). The ORF sequence of Der f 40 was cloned from *D. farinae* total RNA by RT-PCR and verified by DNA sequencing. The Der f 40 ORF cDNA is 321 base pairs long and encodes 106 aa with a theoretical molecular mass of 12.03 kDa (Fig. 2C). The Der f 40 sequence matched that from the predicted Der f genome and was registered in the NCBI library (Genbank No. OM161118.1). These data confirm the presence of a TRX-like Der f 40 homolog in Der f.

### Characterization of rDer f 40

Purified rDer f 40 produced via pET-Der f 40 plasmid expression in *E. coli* BL21(DE3)lysS produced a ∼12-kDa band in SDS-PAGE (Fig. 2D). The properly folded secondary structure rDer f 40 was observed at 25 °C by circular dichroism spectroscopy at 200–260 nm (Fig. 2E). rDer f 40 exhibited spectra typical of a protein comprising predominantly α-helix structures, which was in line with expectation. This rDer f 40 catalyzed the reduction of insulin disulfides in an enzyme activity assay (Fig. 2F), indicating that rDer f 40 was similar to its natural protein and suitable for IgE binding analysis.

### IgE-binding activity of Der f 40 *in vitro*

**Fig. 3:**
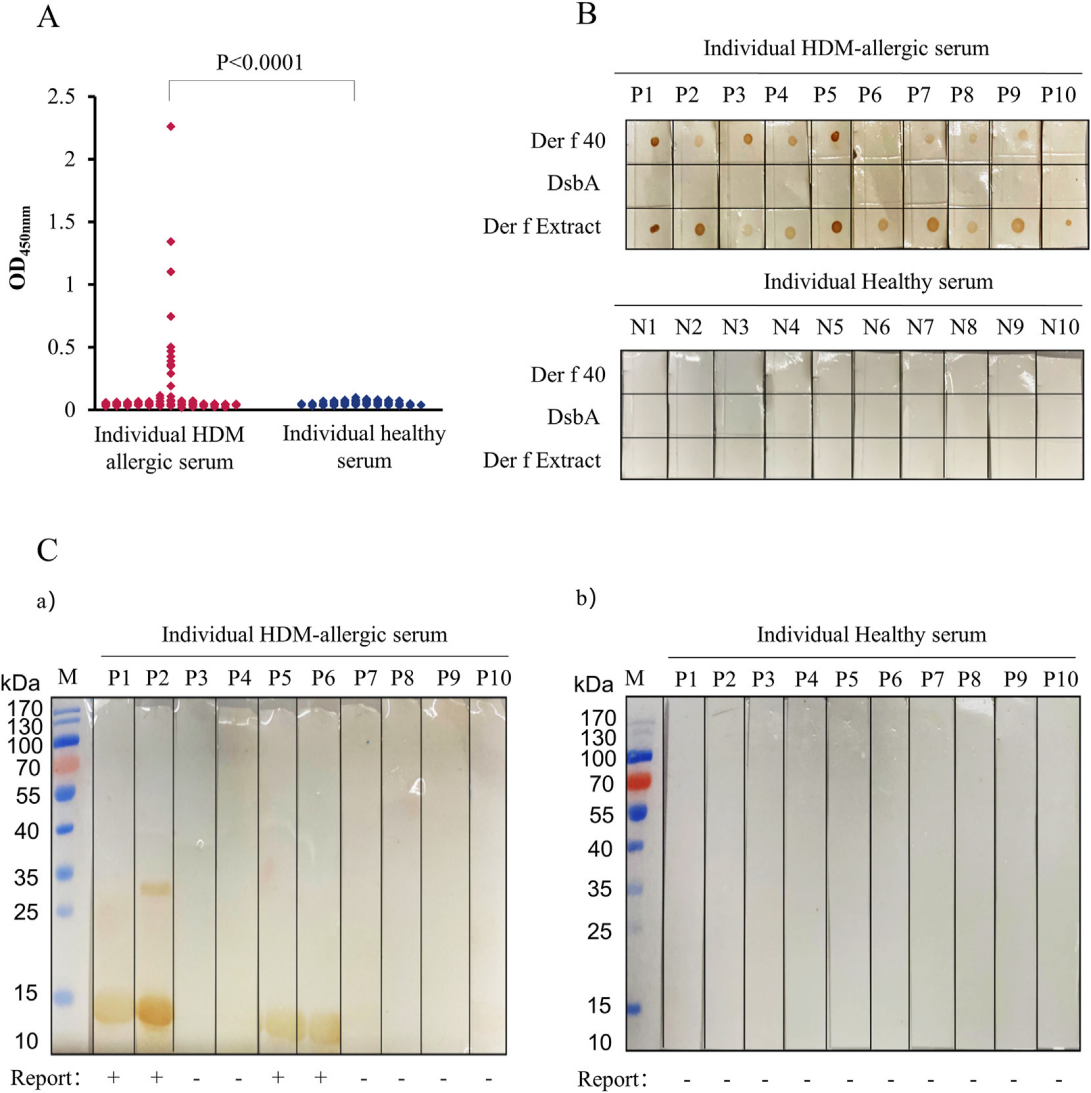
**IgE binding activity of rDer f 40 *in vitro*.** A. IgE binding activity with rDer f 40 shown by IgE-ELISA with HDM allergic sera (124 patient donors) and with healthy control sera (50 control donors). IgE dot-blot (B) and western-blot (C) assays of rDer f 40 with 10 HDM allergic patients' sera and 10 healthy control individuals' sera. The 10 HDM allergic sera were positive in the above mentioned IgE-ELISA assay

The *in vitro* IgE-binding activity to Der f 40 was detected by IgE-ELISA, western-blotting and dot-blotting HDM allergic sera (samples collected from 124 HDM-allergic patients) and healthy control sera (samples collected from 50 individuals without HDM allergies). IgE-ELISA showed that rDer f 40 evoked IgE binding in 9.68% (12/124) of the HDM allergic serum samples (p < 0.0001, Fig. 3A). None of the healthy control serum samples exhibited IgE binding of rDer f 40. Similarly, IgE dot blot (Fig. 3B) and western blot (Fig. 3C) assays showed that rDer f 40 binding occurred selectively with HDM allergic serum IgEs but not with non-allergic serum samples.

### Mast cell activation induced by Der f 40

**Fig. 4:**
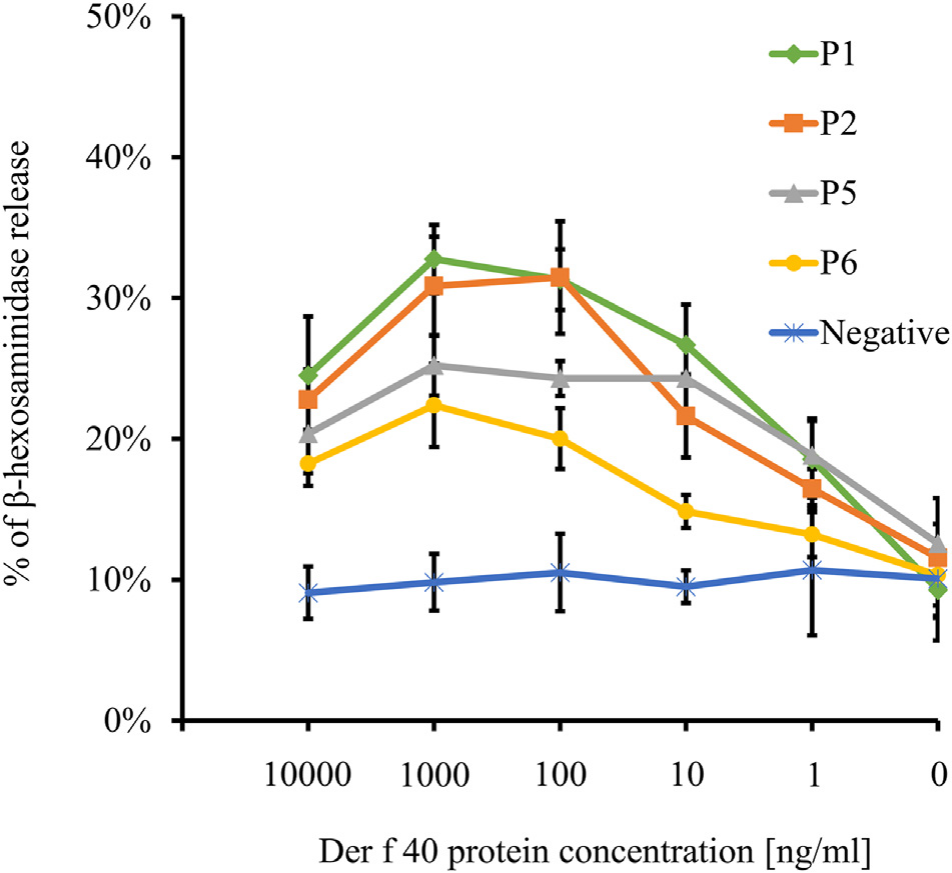
**Mast cell activation induced by rDer f 40.** β-hexosaminidase release from mature BMMCs (from IgE/FcεRI humanized mice) determined after Der f 40 protein stimulation with 4 individual HDM-allergic sera pre-incubated BMMCs, non-HDM allergic healthy control serum

Mast cell activation testing performed with mature BMMCs from humanized-IgE/FcεRI mice showed that rDer f 40 promoted β-hexosaminidase release from BMMCs upon the addition of 4 individual Der f 40-sensitized HDM allergic patients' sera in a dose-dependent manner, thus demonstrating a mast-cell-degranulation effect of rDer f 40 (Fig. 4) and indicating that Der f 40 may cross-link specifically with IgE/FcεRI to activate MC degranulation. In control experiments with non-HDM allergic persons’ serum, no such induction of β-hexosaminidase release was observed. Based on these above results, the WHO/IUIS Allergen Nomenclature Sub-committee listed this protein as Der f 40, a putative novel Der f allergen.

### Immunodominant IgE epitopes of Der f 40

**Fig. 5:**
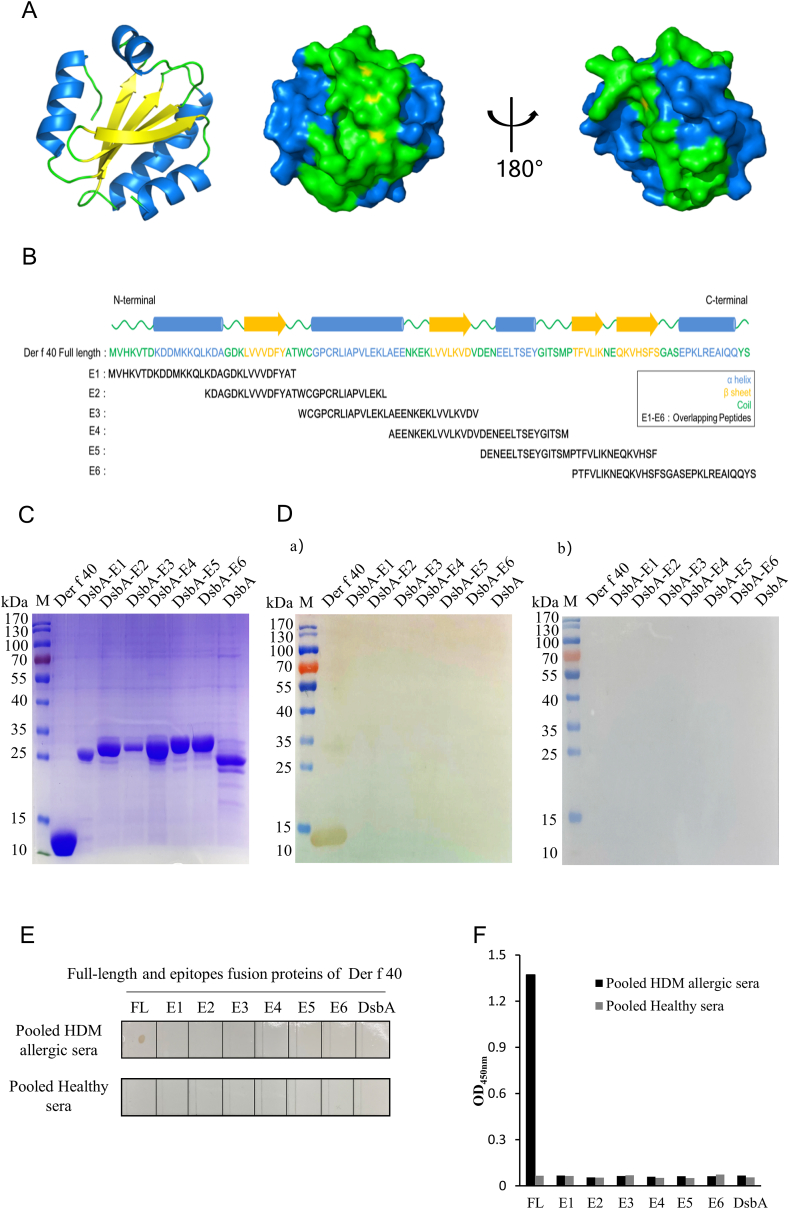
**Screening of truncated Der f 40 proteins for immunodominant IgE binding.** A. Homology model structure of Der f 40 generated using Mala s 13 as a template (PDB code 2J23). B. Schematic representation showing the locations of 6 overlapping epitopes within the secondary structure of Der f 40. C. SDS-PAGE of purified Der f 40 and truncated forms with DsbA fusion proteins expressed in *E. coli*; rDsbA protein was served as a control. IgE western-blot (D), IgE dot-blot (E) and IgE-ELISA with patients' sera pool (N = 10) (a) and healthy control sera pool (N = 10) (b) targeting Der f 40-derived peptide epitope fusion proteins (E1–E6); full-length rDer f 40 protein (FL) was used as a positive control, rDsbA protein was used as a negative control

Homologous 3D structural modeling conducted using Mala s 13 (PDB code 2J23) as a template in SWISS-MODEL software indicated that Der f 40 has 4 α-helices and 4 β-strands (Fig. 5A). Based on the secondary structure analysis of Der f 40, 6 segments of Der f 40 were designed with 30-amino acids length and overlapping by 15 amino acids. These 6 linear epitope regions (E1, aa 1–30; E2, aa 16–45; E3, aa 31–60; E4, aa 46–75; E5, aa 61–90; and E6, aa 76–106) were selected for immunodominant IgE epitope screening (Fig. 5B). Recombinant proteins wherein each of the 6 epitopes regions was fused with DsbA protein were expressed via a pET-DsbA prokaryotic expression system and purified using Ni-NTA resins (Fig. 5C). According to IgE western blot (Fig. 5D), dot blot (Fig. 5E), and ELISA (Fig. 5F) analyses, none of these 6 DsbA fusion proteins were reactive with IgEs from Der f 40-binding sera, indicating that the immunodominant IgE epitope(s) of Der f 40 may be conformational.

### Localization of immunodominant IgE epitopes of Der f 40

**Fig. 6:**
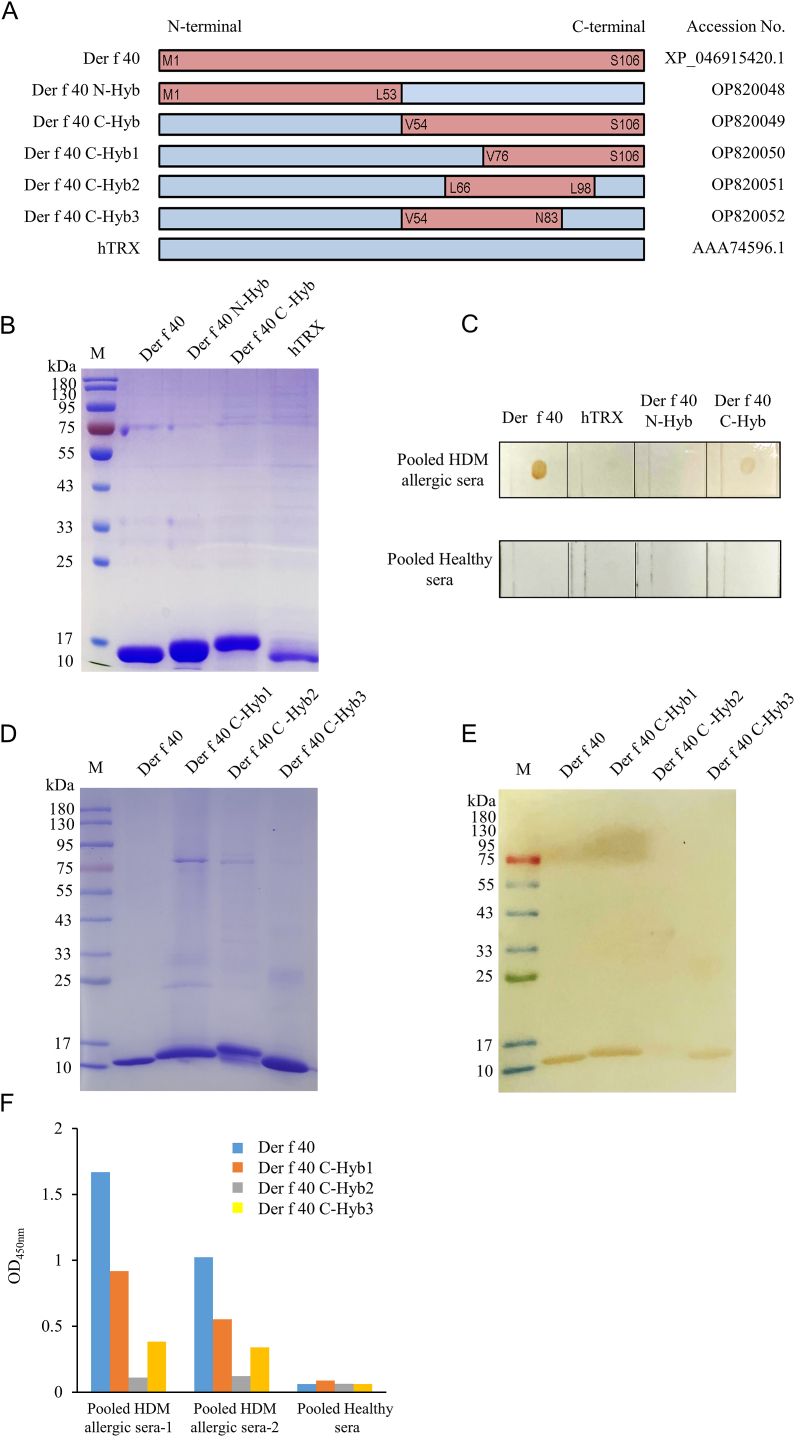
**Identification immunodominant IgE epitope of Der f 40 using hybrid proteins.** A. Schematic diagram of Der f 40 and of Der f 40-human thioredoxin (hTRX) hybrid proteins. B. SDS-PAGE of purified Der f 40, Der f 40 N-terminal hybrid protein (Der f 40 N-Hyb), Der f 40 C-terminal hybrid protein (Der f 40 C-Hyb), and hTRX. C. IgE binding assessment of Der f 40-derived hybrid proteins through IgE-dot-blot with HDM allergic sera and healthy control sera. D. SDS-PAGE of Der f 40 C-terminal hybrid proteins (Der f 40 C-Hyb1, Der f 40 C-Hyb2 and Der f 40 C-Hyb3). E. IgE western-blot with HDM allergic sera targeting Der f 40-derived C-terminal detailed hybrid proteins; full-length Der f 40 protein was used as a positive control. F. IgE binding activity of Der f 40-derived C-terminal detailed hybrid proteins through IgE-ELISA with 2 different pooled HDM allergic patient's serum samples; pooled healthy control sera was used as a negative control

The Der f and human components of purified rDer f 40, hybrid Der f 40 proteins and human Trx protein are presented in Fig. 6A. The synthetic genes Der f 40 N-Hyb, Der f 40 C-Hyb, Der f 40-Hyb1, Der f 40-Hyb2, Der f 40-Hyb3 were registered in the Genbank (accession no. OP820048, OP820049, OP820050, OP820051, OP820052, respectively). These Der f 40-based hybrid proteins were expressed in a pET expression system, purified with Ni-NTA resins, and analyzed by SDS-PAGE (Fig. 6B). The hybrid protein containing the Der f 40 C-terminal region (Der f 40 C-hyb) showed IgE binding with HDM allergic sera (Fig. 6C). To further determine the section of the C-terminal region with an immunodominant IgE epitope, shortened hybrid sequences were constructed (Fig. 6A&D). IgE binding activity assays showed that one such truncated hybrid protein, namely Der f 40 C-hyb1, had robust reactivity to Der f 40-allergic sera (Fig. 6E&F), indicating that the 76–106 region of the C-terminus contains an immunodominant IgE epitope of Der f 40 that is largely conformationally dependent.

## Discussion

In the present study, we generated an rDer f 40 protein that was bound selectively by IgEs from HDM allergic sera and promoted the release of β-hexosaminidase from BMMCs, dose-dependently, when administered with HDM allergic sera. Our rDer f 40 reacted with 12/124 (9.68%) HDM-allergic serum samples. Spectroscopy and enzyme activity assays indicated that our rDer f 40 was similar to the natural Der f Group 40 protein, which was named Der f 40 and listed by the WHO/IUIS Allergen Nomenclature Sub-committee. Immunodominant IgE binding was localized to the aa-76–106 region of the C-terminus of Der f 40.

The present work contributes to the identification of HDM allergens, which is useful for understanding how HDM allergy symptoms are triggered and for allergen immunotherapy development.^19^ The recently identify HDM allergen Der p 37, a 26 kDa allergen with homology to chitin-binding proteins, has been shown to be associated with a heightened risk of allergic asthma.^20^ A study of component-resolved diagnosis of HDM allergens showed that HDM Group 1 allergens constitute a critical sensitizing component for HDM allergies and represent the most clinically relevant allergenic component for effective allergen immunotherapy.^21^ The continuous discovery of HDM allergens broadens our understanding of HDM allergen components which serves to improve the quality of diagnosis and patient care.^22^

We employed allergen sequence homology analysis with the Der f genome to demonstrate the allergenicity of Der f 40. Due to high sequence similarity, allergen homologs can be considered potential allergen candidates. A number of HDM allergens with high aa-sequence homologies, including Der f 4,^23^ Der f 23,^24^ Der p 22,^25^ Der p 24,^26^ Der f 37 and Der f 39,^11^ have been cloned and identified. A higher homology rate does not necessarily indicate a greater likelihood for a protein to be an allergen. The Der f-derived heat shock cognate 70-like protein has 84.83% aa-sequence homology with the *Aedes aegypti* allergen Aed a 8 but elicited no IgE reactivity with HDM allergic sera.^27^ It would be prudent to subject the remaining 21 Der f candidate proteins with >50% homologous aa sequences to further allergenicity probing. Der f 40, one of those 21 remaining homologs, had 52% homology with Plo i 2. Because it is impossible to predict accurately whether a homolog is an allergen based on homology, allergen identification requires that the IgE binding activity of each allergen homolog be determined empirically.

The availability of a chromosome-level Der f genome improved the accuracy of HDM candidate allergen sequences and can facilitate the identification of allergen homologs, as with the identification of Der f 23.^24^ Despite our entrance into the omics era, screening and identification of HDM allergens remains challenging. It is important to obtain an ample pool of candidate proteins for determining allergenic activity. Due to the diversity of candidate allergen characteristics, it is difficult to extract natural candidate proteins.^3^ Thus, it is useful to use recombinant proteins expressed in *E. coli* or yeast expression systems.^28^^,^^29^ However, it is necessary to note that protein misfolding in *E. coli* can lead to the production of insoluble proteins that will require biochemical refolding to restore full IgE-binding and/or enzymatic activity. When a recombinant protein is folded into its correct native structure, it can reflect the IgE binding activity of a candidate homolog. The eventual recombinant proteins production of the 21 potential homolog candidates, which will be a challenge to achieve, could enable the discovery of novel HDM allergens. Only 10 in the 21 remaining homologs have been obtained with soluble recombinant protein in an *E. coli* expression system (data not shown).

Currently, basophil/mast cell activation assays are the most frequently used tools to verify an allergic response.^30^ Following exposure to external allergens, the aggregation and cross-linkage of IgE to its high-affinity receptor FcεRI complexes results in mast cell degranulation and release of pro-inflammatory mediators responsible for perpetuating allergic inflammatory disorders.^31^ Traditionally, mast cells were generated from peripheral blood progenitors.^32^ However, there is a practical difficulty in obtaining a quantity of purified primary cells from patients that will be sufficient for research purposes.^30^ The usage of mature BMMCs from humanized IgE/FcεRI mice together with allergy patient sera provides a solution to this problem. Using this approach, we showed in this study that rDer f 40 promoted the release of β-hexosaminidase from BMMCs in the presence Der f 40-sensitized HDM allergic patients’ sera.

The hybrid proteins method provides a straightforward way to screen for allergen epitope regions of conformational IgE epitopes. It requires IgE antibodies from allergic patient-derived sera or artificially prepared monoclonal antibodies. Unlike sequential overlapping peptides methods, it has the advantage of screening conformational IgE epitopes.^12^^,^^14^ The results we obtained in this study with the hybrid proteins method showed that the IgE-binding epitope of Der f 40 is likely formed by the three-dimensional conformational structure of the C-terminal region of the allergen. The Der f 40-derived hybrid proteins we designed were all soluble to be expressed in *E. coli*. In general, the soluble recombinant proteins of Der f mite usually may have correct folding to maintain IgE-binding capacity according to our studies.^10^, ^11^, ^12^ In our current study, the recombinant Der f 40 protein had relative corrected structure and maintained the IgE-binding capacity. The structure of hybrid proteins should be similar to that of Der f 40 due to their high amino acid sequence homology. In addition, the main aim of the study was to show the immunodominant IgE epitopes of Der f 40. In fact, the hybrid protein 1 was stronger than hybrid protein 2 to bind specific IgE. We have reported the clear data. However, whether hybrid protein 2 contained IgE epitopes needs further experimental confirmation. Using the same method previously, we were able to locate the immunodominant IgE binding epitopes of Der f 24^14^ and Der p 39.^12^

HDM Group 40 allergens belong to the TRX family of proteins. TRX is a redox-active protein that regulates reactive oxidative metabolism and plays a crucial antioxidant role, regulating the reduction/oxidation balance by scavenging reactive oxygen species.^33^ The TRX homolog Plo i 2 has previously been shown to induce classical Th2-biased immune responses in mice.^34^ In the future, it will be prudent to determine whether Der f 40 affects the activation of macrophages, epithelial cells or other functional cells in Der f 40 induced allergic inflammation.

## Conclusion

Der f 40, a novel HDM allergen, was identified. The immunodominant IgE binding epitope of Der f 40 appears to be conformational in nature and to be located mainly in the C-terminal region of the allergen. This study can provide reference information relevant for HDM allergy diagnosis and specific immunotherapy development.

## Abbreviations

HDM, house dust mite; Der f, *Dermatophagoides farinae*; Der p, *Dermatophagoides pteronyssinus*; Der f 40, Group 40 allergen of *Dermatophagoides farinae*; Der p 40, Group 40 allergen of *Dermatophagoides pteronyssinus*; BMMCs, Bone Marrow Mononuclear Cells; TRX, Thioredoxin; IgE, immunoglobulin E; ELISA, Enzyme-linked immunosorbent assay; IPTG, Isopropyl-β-d-thiogalactopyranoside; HRP, Horseradish peroxidase.

## Acknowledgments

We thank Miss Yan Zhang from Ji Kunmei lab for her technologic support in the protein expression and purification and other members of Ji Kunmei lab for their critical comments in the manuscript preparation.

## Funding

The present study was supported in part by research funding from the 10.13039/501100001809National Natural Science Foundation of China (grant no. 82071806), Guangdong Province (grant no. 2021A1515011140), and 10.13039/501100004791Shenzhen City (grant no. JCYJ20210324095004012 and JCYJ20190808155603545).

## Author contributions

CZL, LS and LWY performed experiments and interpreted results. CZL, ZZW and HWZ contributed to the data analysis. JJC and KJ supervised the projects and participated in experimental design and technical discussions. CZL and CJJ wrote the paper. KJ revised the manuscript.

## Availability of data and materials

The datasets used and/or analyzed during the current study are available from the corresponding author on reasonable request.

## Ethics approval and consent to participate

Permission to conduct this study was obtained from the Ethics Committee of the First Affiliated Hospital of Guangzhou Medical College (No. 2012-51). Informed consent was obtained from all individual participants included in the study. All procedures involving human participants were in accordance with the ethical standards of the committee.

## Authors’ consent for publication

I confirm that each of the authors has reviewed this paper in its submitted form and approved submission for publication of this paper to the World Allergy Organization Journal.

## Declaration of competing interest

The authors declare no competing interests.
